# ﻿Two new Palaearctic species of *Xynobius* Foerster (Hymenoptera, Braconidae, Opiinae)

**DOI:** 10.3897/zookeys.1160.103417

**Published:** 2023-05-03

**Authors:** Yunjong Han, Cornelis van Achterberg, Heung-Sik Lee, Hyojoong Kim

**Affiliations:** 1 Animal Systematics Laboratory, Department of Biological Science, Kunsan National University, Gunsan, 54150, Republic of Korea Kunsan National University Gunsan Republic of Korea; 2 Naturalis Biodiversity Center, P.O. 9517, 2300 RA Leiden, Netherlands Naturalis Biodiversity Center Leiden Netherlands; 3 Animal and Plant Quarantine Agency, Gimcheon, 39660, Republic of Korea Animal and Plant Quarantine Agency Gimcheon Republic of Korea

**Keywords:** Japan, key, new species, Norway, parasitoid, setose mesoscutum

## Abstract

Two new and very similar species of the genus *Xynobius* Foerster, 1863 are described and illustrated, *X.subparallelus* Han & van Achterberg, **sp. nov.** from Japan (Honshu) and *X.setosiscutum* van Achterberg, **sp. nov.** from Norway. Three species are newly reported from Norway: *Xynobiusaciculatus* (Thomson, 1895), *X.comatus* (Wesmael, 1835), and *X.polyzonius* (Wesmael, 1835). *X.polyzonius* (Wesmael, 1835) and *X.sapporanus* (Fischer, 1963) are new combinations. Identification keys to the *Xynobius* species known from Norway and Japan are added.

## ﻿Introduction

Opiinae is a large subfamily of the family Braconidae with approximately 2,000 valid species and 39 genera according to Yu et al. (2016). It is a common group of parasitoid wasps containing mainly mining or fruit-infesting dipterous larvae and has a worldwide distribution. Wharton (e.g. 1987, 1988, 1997) published important updates and some additions for the existing keys to the genera of Opiinae, but the number of genera remains a matter of discussion because the limits of some genera, especially of *Opius* Wesmael, 1835 and *Eurytenes* Foerster, 1863, are uncertain. We follow Li et al. (2013) and treat the genus *Xynobius* Foerster, 1863 as a valid genus separate from *Opius* Wesmael, 1835, not included within it as was done in the past.

During a visit to Osaka Museum of Natural History the first author discovered a remarkably setose species from Japan (Honshu), and the second author discovered a similar species from south-west Norway among Malaise-trap material. These new taxa are compared, described, and illustrated below.

## ﻿Material and method

The Japanese specimen was collected by using a sweep net. The Norwegian specimens were collected in a Malaise trap and were chemically treated with a mixture of xylene + alcohol 96% and amylacetate (AXA-method; van Achterberg 2009). For identification of the subfamily Opiinae, see van Achterberg (1990, 1993, and 1997); for references to the Opiinae, see Yu et al. (2016).

Morphological terminology follows van Achterberg (1988, 1993), including the abbreviations for the wing venation. Measurements are taken as indicated by van Achterberg (1988); for the length and the width of a body part the maximum length and width is taken, unless otherwise indicated. The length of the mesosoma is measured from the anterior border of the mesoscutum up to the apex of the propodeum and of the first tergite from the posterior border of the adductor up to the medio-posterior margin of the tergite.

Observations, photographic images, and descriptions were made either under a digital stereo microscope (VHX-1000, Keyence) or with a Canon 5Ds 50.6-megapixel camera combined with a Canon MP-E 65 mm f/2.8 1–5× macro lens, Laowa KX-800 macro twin flash, and an electronic WeMacro Z-stepper rail. The photos were stacked with Helicon Focus v. 7 software (HeliconSoft, Kharkiv, Ukraine).

The type specimens are deposited in the Osaka Museum of Natural History (**OMNH**) at Osaka, Naturalis Biodiversity Center (**RMNH**) at Leiden and Museum Stavanger (MSC) at Stavanger.

## ﻿Systematics

### ﻿Genus Xynobius

Taxon classificationAnimaliaHymenopteraBraconidae

Foerster, 1863

90E205DB-7D8C-53CF-9C9E-B2D2A711DF63

Figs 1 , 2–11

Xynobius Foerster, 1863: 235. Type species (by original designation): Xynobiuspallipes Foerster, 1863 (= Opiuscaelatus Haliday, 1837).
Aclisis Foerster, 1863: 267. Type species (by original designation): Aclisisisomera Foerster, 1863 (= Opiuscaelatus Haliday, 1837). Synonymized by Fischer (1972).
Holconotus Foerster, 1863: 259 (not Schmidt-Göbel 1846). Type species (by original designation): Opiuscomatus Wesmael, 1835). Synonymized by van Achterberg (2004).
Aulonotus Ashmead, 1900: 368 (new name for Holconotus Foerster). Type species (by original designation): Opiuscomatus Wesmael, 1835). Synonymized by Tobias and Jakimavicius (1986).
Eristernaulax Viereck, 1913: 362. Type species (by original designation): Eristernaulaxleucotaenia Viereck, 1913). Synonymized by van Achterberg (2004).
Stigmatopoea Fischer, 1984: 610, 611 (as subgenus of Opius Wesmael), 1998: 25 (key to species); Wharton 1988: 356; 2006: 338 (as subgenus of Eurytenes Foerster, 1863; possible paraphyly in Xynobius). Type species (by original designation): Opiusmacrocerus Thomson, 1895. Synonymized by van Achterberg (2004).
Xynobiotenes Fischer, 1998: 23 (as subgenus of Eurytenes Foerster, 1863). Type species (by original designation): Opiusscutellatus Fischer, 1962. Synonymized by Li et al. (2013).

### ﻿ Xynobius subparallelus

Taxon classificationAnimaliaHymenopteraBraconidae

Han & van Achterberg sp. nov.

0B9688BC-77A1-583F-A1F1-B2EF4666AEF5

https://zoobank.org/ED84A031-AD98-4616-943A-9895E5BF4AF6

#### Type material.

***Holotype***, ♀ (OMNH), “Japan: Naihara, Totsukawa, Yoshino District, 34°05'49"N, 135°52'20"E, 11.viii.2013, SW [= collected by sweeping], Shunpei Fujie, OMNH”

#### Diagnosis.

This species belongs to the *Xynobiuscomatus* group on account of the evenly and conspicuously setose middle lobe of the mesoscutum and scutellum (Figs 3, 4), but it differs from all other species by the subparallel-sided first tergite (Fig. 5; about 1.8× longer than its apical width), short temple (Fig. 8; eyes in dorsal view about 2.1× longer than temple), irregularly and weakly striate second tergite (Fig. 6), and vein m-cu of the fore wing that gradually merges into vein 2-CU1 (Fig. 2, but this character is rather variable in *X.setosiscutum*). In addition, the notauli are largely absent on the mesoscutal disc (Fig. 4, a derived character state in common with *X.setosiscutum* sp. nov. from Norway), and the second tergite is longitudinally striate (Fig. 6).

**Figure 1.**
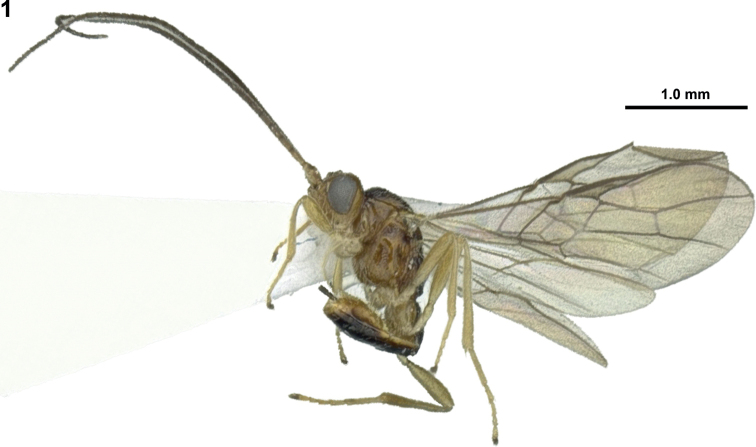
*Xynobiussubparallelus* Han & van Achterberg, sp. nov., holotype, ♀, Japan, habitus, lateral.

**Figures 2–11.**
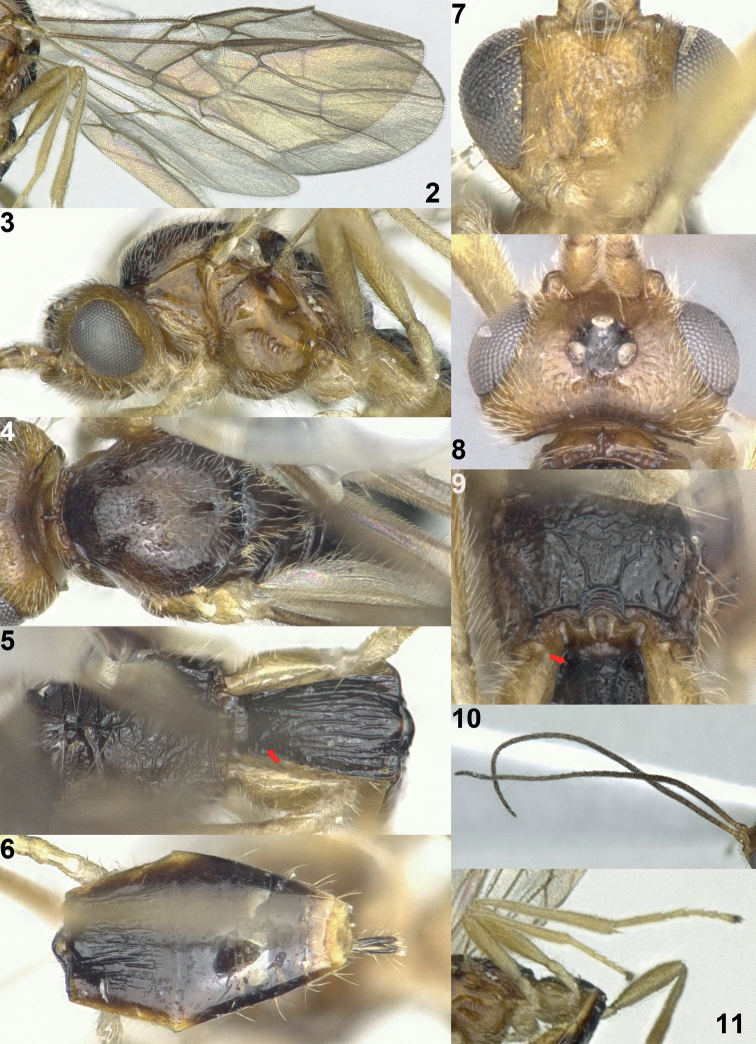
*Xynobiussubparallelus* Han & van Achterberg, sp. nov., holotype, ♀, Japan **2** wings **3** mesosoma lateral **4** mesosoma dorsal **5** propodeum and 1^st^ metasomal segment dorsal **6** 2^nd^ and following metasoma segments dorsal **7** head anterior **8** head dorsal **9** propodeum posterior and 1^st^ metasomal segment basal **10** antenna **11** hind leg lateral. The arrow indicates the dorsope.

#### Description.

Female; length of body 2.7 mm, of fore wing 2.6 mm and of antenna about 3.4 mm.

***Head*.** Antenna with 33 segments (Fig. 10), 1.2× longer than body; margin of antennal sockets strongly protruding, depression between antennal sockets (Fig. 7); length of eye in dorsal view 2.1× longer than temple (Fig. 8); height of head 1.35× longer than height of eye; vertex and frons punctate, setose except for large, smooth interspaces on vertex; no median keel on frons (Fig. 7); width of clypeus twice longer than its maximum height; hypoclypeal depression large (Fig. 7); length of the maxillary palp 1.4× longer than height of head; malar sulcus absent; occipital carina absent dorsally; mandible robust (Fig. 1), symmetric, gradually widened basally.

***Mesosoma*.** Length of mesosoma 1.4× longer than its height (Fig. 3); pronope absent but with transverse crenulated groove (Fig. 4); mesopleuron largely smooth, but precoxal sulcus medially impressed and coarsely crenulate (Fig. 3); mesopleural sulcus largely smooth; notauli absent on disc except for a pair of short, deep impressions anteriorly (Fig. 4); mesoscutum and scutellum shiny, punctulate, and densely setose; medio-posterior depression of mesoscutum round and rather small (Fig. 4); scutellar sulcus medium-sized and distinctly crenulate; scutellum flat and only posteriorly narrowly sculptured; propodeum reticulate-rugose, with short medio-longitudinal carina anteriorly but posteriorly largely smooth between carinae (Figs 5, 9).

***Wings*.** Fore wing (Fig. 2): pterostigma narrow elliptical, gradually narrowed apically; vein 1-SR+M sinuate; vein 2-SR distinctly oblique; vein 3-SR 1.7× longer than vein 2-SR; vein SR1 slightly curved; r:3-SR:SR1 = 4:26:46; vein m-cu distinctly antefurcal; second submarginal cell elongated; first subdiscal cell transverse and elongated (Fig. 2). Hind wing: vein m-cu oblique and only pigmented; vein 1r-m 0.5× as long as vein 1-M.

***Legs*.** Length of hind femur 5.3× longer than its width (Fig. 11).

***Metasoma*.** Length of first tergite 1.8× longer than its apical width, its surface rugose with longitudinal striae and rather flat in lateral view (Fig. 5); dorsope distinctly present (Figs 5, 9); second tergite finely and irregularly longitudinally striate medially (Fig. 6) and distinctly longer than third tergite; second metasomal suture absent (Fig. 6); following tergites smooth and with few setae posteriorly; length of setose part of ovipositor sheath 0.5× longer than length of first tergite and nearly 0.1× as long as fore wing, slightly protruding beyond apex of metasoma (Fig. 1).

***Colour*.** Generally dark brown dorsally (Fig. 1); head, scape, mesopleuron and pronotum, yellowish brown; legs and palpi, pale brownish yellow.

#### Distribution.

Japan (Honshu).

#### Biology.

Unknown.

#### Etymology.

Named after the subparallel-sided first metasomal tergite; “*sub*” is Latin for “less than” and “*parallelus*” is Latin for “equidistantly sides”.

#### Remarks.

The new species has a distinct dorsope, symmetric mandible, vein r much shorter than vein 2-SR and a large hypoclypeal depression; therefore, it belongs to the genus *Xynobius*. Most important is the slender (subparallel-sided) first metasomal tergite, the irregularly and weakly longitudinally striate second tergite, the entirely setose mesoscutum and the reduced notauli (absent on most of mesoscutal disc and only distinct and crenulate anteriorly). In the key by Tobias (1998), this species runs to the subgenus Apodesmia Foerster sensu Tobias and (surprisingly) to O. (Opius) angusticellularis Tobias, 1998. This species has little to do with the new species because the mesosoma is only slightly longer than high in lateral view, the second and third metasomal tergites are granulate and the antenna has 22–24 segments. The new species runs in Chen and Weng (2005) to Opius (Apodesmia) isabella Chen & Weng, 2005, but it belongs to the genus *Apodesmia* Foerster, 1863 because the occipital carina is connected to the hypostomal carina ventrally, the second and third tergites are more or less coriaceous, and the clypeus is only 1.2× wider than long. Actually, the new species is similar to *Xynobiuswengi* van Achterberg & Li, 2013 because of the setose mesoscutum and scutellum and striate second metasomal tergite. However, *X.wengi* has the crenulate notauli present on the mesoscutal disc (only apical quarter absent; notauli nearly entirely absent on disc in *X.subparallelus*), vein m-cu of the fore wing postfurcal or subinterstitial (distinctly antefurcal in *X.subparallelus*), the first metasomal tergite about 1.3× longer than wide apically (about 1.8× in *X.subparallelus*), and the second tergite regularly and coarsely striate (irregularly and finely striate *X.subparallelus*).

### ﻿ Xynobius setosiscutum

Taxon classificationAnimaliaHymenopteraBraconidae

van Achterberg sp. nov.

53A718FD-9A4B-5069-84F8-8889B2FD24F6

https://zoobank.org/4CEA8E68-639C-4AD0-AEFD-041D92A8708C

Figs 12 , 13–23

#### Type material.

***Holotype***, ♀ (RMNH), “Norway: RY, Sokndal, Skittmyr, 58.3509°N, 6.3054°E, 20.vii.–8.viii.2020, MT [= Malaise trap], J. Birkeland, RMNH’21”. ***Paratypes*** (5): 1 ♂ (RMNH), topotypic, but 10–20.vii.2020; 1 ♀ (MSC), “Norway: RY, Ra, Hølland, 58.5245°N, 5.8352°E, 29.vi. –16.vii.2020, MT, A.T. Mjøs, RMNH’21”; 1 ♀ (RMNH), “Norway: RY, Time, Mossige, 58.6900N 5.7239E, 17.ix.–11.x.2020, MT, A.T. Mjøs, RMNH’21”; 1 ♀ (RMNH), “Norway: RI, Hjelmeland, 59.2312°N, 6.1653°E, 16.ix.–31.x.2020, MT, A.T. Mjøs, RMNH’21”; 1 ♀ (RMNH), “Norway: ROY, Sokndal, Rekvei, Long. lat. 58.2035°N, 6.1559°E, Malaise trap, 7.ix.2019, J. Birkeland”.

#### Diagnosis.

**Figure 12.**
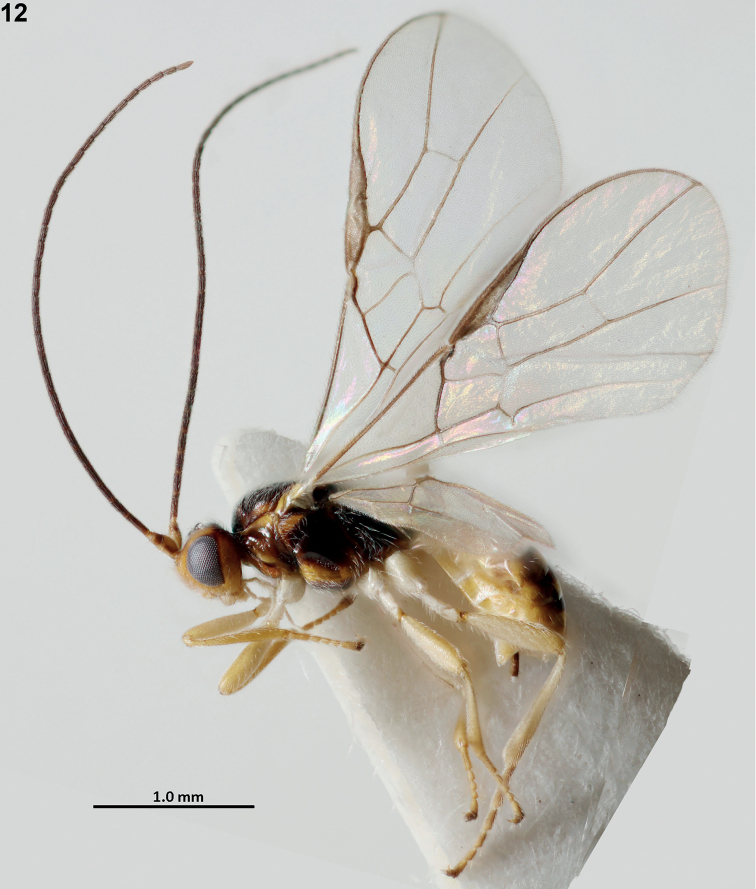
*Xynobiussetosiscutum* van Achterberg, sp. nov., holotype, ♀, Norway, habitus, lateral.

**Figures 13–23.**
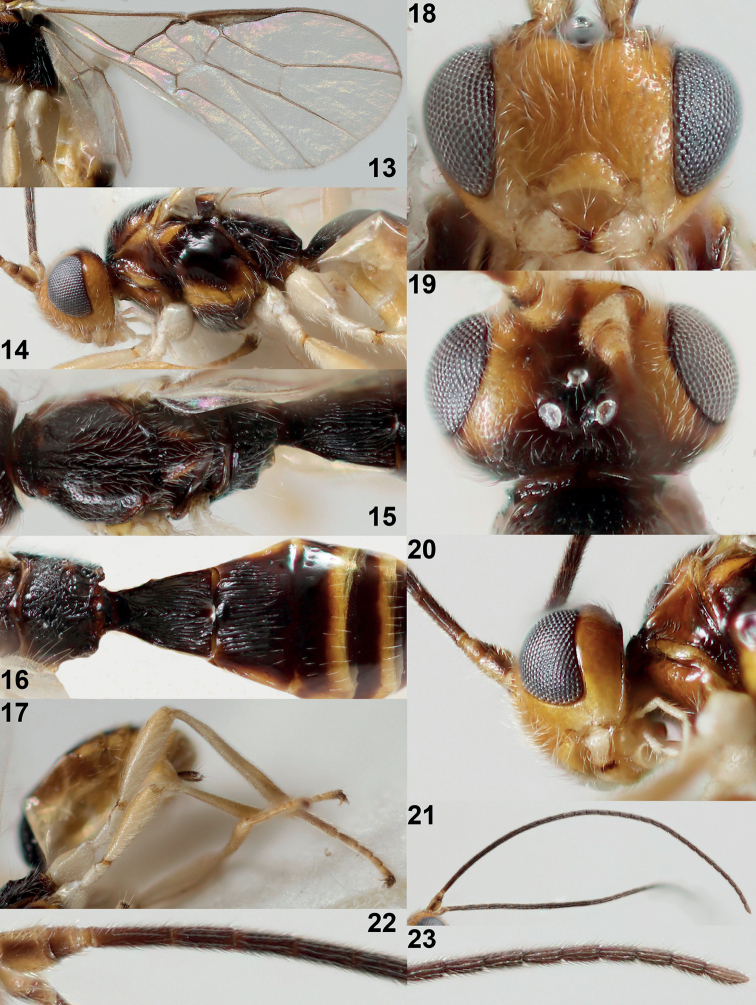
*Xynobiussetosiscutum* van Achterberg, sp. nov., holotype, ♀, Norway **13** wings **14** head and mesosoma lateral **15** mesosoma dorsal **16** propodeum and 1^st^ to 4^th^ metasomal segments dorsal **17** hind leg and metasoma lateral **18** head anterior **19** head dorsal **20** mandible latero-ventral **21** antenna **22** base of antenna **23** apex of antenna.

Antenna with 32–34 segments, flagellum dark brown but apical segments more or less brown; temple medium-sized (Fig. 19; roundly narrowed and eye in dorsal view 2.6× longer than temple); mesoscutum and scutellum evenly and conspicuously setose (Fig. 15); notauli largely absent on mesoscutal disc (a derived character state in common with *X.subparallelus* sp. nov. from Japan); hind femur comparatively robust (Figs 12, 17; about 4× longer than wide); pterostigma narrow elliptical and gradually narrowed apically (Fig. 13); vein m-cu of fore wing distinctly antefurcal and posteriorly angulate with vein 2-CU1 (Fig. 13); first tergite distinctly widened posteriorly (Fig. 16; 1.2–1.4× longer than its apical width in ♀, about 1.6× longer in ♂); second tergite regularly and costate-like striate (Fig. 16) and third tergite smooth; setose part of ovipositor sheath shorter than first metasomal tergite (Fig. 17) and at most slightly protruding beyond apex of metasoma. The new species is very similar to *X.subparallelus* sp. nov. from Japan because of the reduction of the notauli and the conspicuous setosity of the mesoscutum and scutellum. However, it differs by having the first tergite distinctly widened posteriorly (subparallel-sided in *X.subparallelus*), distinctly wider temple (comparatively narrow), apex of third and fourth metasomal tergites yellow (blackish or dark brown), second tergite regularly and coarsely striate (finely and irregularly striate) and hind femur less slender, about 4× longer than wide (more robust, about 5× longer than wide).

#### Description.

Holotype, ♀, length of body 3.0 mm, of fore wing 3.3 mm.

***Head*.** Antenna with 34 segments and 1.2× as long as fore wing; third segment 1.4× longer than fourth segment, length of third, fourth and penultimate segments 4.7×, 3.3×, and 2.5× their width, respectively (Figs 21, 22); width of head 1.8× its median length in dorsal view; no depression behind stemmaticum; vertex flattened and punctulate; OOL: diameter of ocellus: POL= 37:15:22 (Fig. 19); frons largely flattened and setose (Fig. 18); face finely punctate, shiny and with conspicuously long setae (Fig. 18); clypeus convex dorsally, semi-circular, largely smooth (except punctulation because of very long setae) and its ventral margin thick and concave, width of clypeus 2.1× its maximum height and 0.5× minimum width of face; hypoclypeal depression large and deep (Fig. 18); eye in dorsal view 2.6× longer than temple and temple behind eye roundly narrowed (Fig. 19); occipital carina distinct but dorsally finer and medio-dorsally absent (Fig. 19); temple and malar space smooth; length of malar space 0.8× basal width of mandible and 0.2× height of eye; malar suture nearly complete, shallow; mandible slightly twisted apically, both teeth robust, basally symmetric or nearly so, basal half with lamelliform ventral carina (Figs 18, 20); length of maxillary palp 1.3× height of head; labial palp segments elongate.

***Mesosoma*.** Length of mesosoma 1.5× its height (Fig. 14); laterally pronotum smooth only anteriorly, medially and posteriorly with few crenulae; dorsal pronope absent, medial area rather short, laterally with narrow groove; propleuron weakly evenly convex, with long setae, shiny and smooth (Fig. 14); mesopleuron smooth except for coarsely crenulate precoxal sulcus medially (Fig. 14); mesosternum densely setose; postpectal carina absent; pleural sulcus smooth or nearly so; mesosternal sulcus narrow and finely crenulate; metapleuron largely smooth dorsally and ventrally rugulose, long setose (Fig. 14); mesoscutum steeply raised above pronotum, densely setose, rather shiny and punctulate; notauli short, only impressed anteriorly and absent on most of mesoscutum, rather deep and largely smooth (Fig. 15); medio-posterior depression of mesoscutum rather deep medially, linear and medium-sized; transverse suture of mesoscutum present; scutellar sulcus deep and broad medially, with four carinae and medially 0.2× as long as scutellum; scutellum largely smooth and setose, punctulate, weakly convex, with narrow subposterior depression (Fig. 15); side of scutellum partly punctate (Fig. 15); propodeum largely vermiculate-rugose but posteriorly largely smooth between carinae, anteriorly with short medio-longitudinal carina (Figs 15, 16).

***Wings*.** Fore wing (Fig. 13): pterostigma elongate-elliptical, 5× as long as its maximum width and gradually merging into vein 1-R1; vein M+CU1 weakly curved and only distal quarter sclerotized; 1-R1 reaching wing apex; r:3-SR:SR1 = 4:45:72; 2-SR:3-SR:r-m = 20:45:16; vein r slightly widened, its length 0.3× width of pterostigma, arising far before middle of pterostigma; 2-SR straight; m-cu distinctly antefurcal, largely unpigmented and slightly curved, angled with 2-CU1; cu-a slightly postfurcal and vertical; 1-CU1 widened; vein 3-CU1 distinctly longer than vein CU1b (Fig. 13). Hind wing: M+CU:1-M:1r-m = 20:21:12; cu-a straight; m-cu present.

***Legs*.** Second to fourth fore tarsal segments hardly longer than wide; hind femur, tibia and basitarsus 4.0×, 9.6×, and 5.3× as long as wide, respectively (Fig. 17); hind femur densely and long setose.

***Metasoma*.** First tergite 1.2× as long as wide apically and slightly widened apically, dorsope rather small, its surface convex medially and largely coarsely striate, dorsal carinae distinct in basal third of tergite (Fig. 16); second tergite regularly costate-like striate and following tergites smooth; second suture absent dorsally, except laterally; setose part of ovipositor sheath 0.04× as long as fore wing (entire sheath 0.06×), 0.3× first tergite, and 0.1× as long as hind tibia; sheath slightly protruding beyond apex of metasoma; hypopygium truncate ventro-apically, membranous medially and about 0.6× as long as first tergite (Fig. 17).

***Colour*.** Black or blackish brown; scape and pedicel largely (but dorsally partly dark brown), mandible, palpi, coxae, trochanters and trochantelli and femora basally ivory or whitish; hind tarsus infuscate; remainder of legs, head except occiput, vertex and frons medially, mesoscutum antero-laterally, scutellum laterally, pronotum partly, mesopleuron antero-dorsally and ventrally, third to fifth tergites apically, sixth and seventh tergites, and metasoma ventrally yellow; scutellum mainly dark reddish brown; pronotum medially and propleuron, pterostigma, and most veins brown; antenna dark brown, ventrobasally yellowish, apically nearly brown; ovipositor sheath dark brown; wing membrane subhyaline (Fig. 13).

***Variation*.** Length of body 2.9–3.1 mm, of fore wing 3.2–3.5 mm (of ♂ 2.9 mm); antennal segments in ♀ 32 (2) and 34 (2) and in ♂ 33 (1); flagellum dark brown or brown; mesoscutum posteriorly entirely black or partly brown and medio-posterior depression droplet-shaped or linear; vein m-cu of fore wing angled with vein 2-CU1 or gradually merging into vein 2-CU1; hind femur 4.0–4.2× longer than wide; first tergite 1.2–1.4× longer than wide apically in ♀ (about 1.6× longer in ♂); length of setose part of ovipositor sheath 0.03–0.04× fore wing (exposed sheath 0.06–0.08×).

#### Distribution.

Southwestern Norway.

#### Biology.

Unknown.

#### Etymology.

Named after the entirely and conspicuously setose mesoscutum; “*setosus*” is Latin for “bristly”, and “*scutum*” is Latin for “shield”.

#### Remarks.

This species runs to the subgenus Allotypus Foerster sensu Fischer, and with difficulty to *Opiussaevulus* Fischer, 1958 (mesosoma less than 1.5× longer than high in lateral view) or *O.irregularis* Wesmael, 1835 (mesosoma 1.5× longer than high in lateral view), in the keys by Fischer (1972). Both of these species have nothing in common with the new species and both belong to the genus *Apodesmia* Foerster, 1863 because the occipital carina is curved and connected to hypostomal carina. Actually, the new species is more related to *X.aciculatus* (Thomson, 1895) because they share the setose middle lobe of the mesoscutum, the striate second tergite, the (at least partly) yellow face and clypeus, and the comparatively robust first tergite in females. The new species has the lateral mesoscutal lobes largely setose medially (glabrous in *X.aciculatus*); vein 3-CU1 of fore wing distinctly longer than vein CU1b (about of equal length); vein r of fore wing widened and shorter (narrow and longer); antenna of ♀ with 32–34 segments (with 28–31 segments); sixth metasomal tergite yellow largely dark brown); second tergite coarsely striate (finely striate); notauli largely absent on mesoscutal disc (notauli complete on disc); and vein m-cu of fore wing antefurcal (postfurcal).

### ﻿Key to Norwegian species of the genus *Xynobius* Foerster

**Notes.** The following species are new for Norway and based on material received from Jarl Birkeland and Alf Tore Mjøs (RMNH). *Xynobiusaciculatus* (Thomson): RY, Sokndal, Skittmyr; RI, Hjelmeland; *X.comatus* (Wesmael): RI, Suldal, Skumpanes; *X.polyzonius* (Wesmael): RY, Sokndal, Skittmyr. The new combination is based on the examination of the type series.

**Table:** 

1	Temples and face densely punctate; scutellum densely rugose; pronotal side (except dorsally) extensively rugose; hind coxa rather dull and densely sculptured; [antenna with about 50 segments; clypeus strongly protruding forwards]	***X.caelatus* (Haliday, 1837)**
–	Temples smooth or nearly so; face at most remotely punctate; scutellum smooth or largely so; pronotal side (except medial and posterior grooves) smooth or nearly so; hind coxa shiny and smooth or sparsely punctulate	**2**
2	Pterostigma behind vein r subparallel-sided or slightly concave; [= “*Stigmatopoea* Fischer, 1986”]; [notauli on mesoscutal disc largely absent posteriorly; antenna with 46–57 segments]	***X.macrocerus* (Thomson, 1895)**
–	Pterostigma behind vein r slightly to strongly narrowed (Fig. 13)	**3**
3	Precoxal sulcus smooth or granulate; malar suture (rather) deep; head (except more or less clypeus) blackish or dark brown; propodeum without a distinct median carina anteriorly or weakly developed; [notauli largely absent on disc]	**4**
–	Precoxal sulcus distinctly crenulate(-rugose) submedially; malar suture absent, very short or shallow; head partly or largely brownish yellow; propodeum with a distinct median carina anteriorly or a pentagonal areola medially	**5**
4	Antennal segments of ♀ 38–42; area below pterostigma with brownish patch, rarely obsolescent; vein M+CU1 of fore wing largely sclerotized; [fourth antennal segment robust; propleuron crenulate posteriorly]	***X.geniculatus* (Thomson, 1895)**
–	Antennal segments of ♀ 26–35; area below pterostigma hyaline; basal half of vein M+CU1 of fore wing unsclerotized; [basal antennal segments comparatively stout and dark brown]	***X.maculipes* (Wesmael, 1835)**
5	Second metasomal tergite completely smooth **and** notauli largely absent on disc of mesoscutum; pronope large to medium-sized and deep; mandible without ventro-basal carina; second metasomal tergite yellowish or yellowish brown; basal half of vein M+CU1 of fore wing sclerotized; eyes of ♀ nearly touching mandibular condyle because of short malar space	***X.polyzonius* (Wesmael, 1835) comb. nov.**
–	Second tergite coarsely striate **or** notauli at least present on anterior half of mesoscutal disc; pronope small, obsolescent or absent; mandible with a short ventro-basal carina; second tergite blackish or dark brown; basal half of vein M+CU1 of fore wing unsclerotized; eyes of ♀ remain distinctly removed from mandibular condyle because of moderately long malar space	**6**
6	Antenna of ♀ with 22–24 segments, at most 1.2× longer than body; apex of metasoma of ♀ dark brown; hind femur at least partly very finely and densely sculptured and with long setae dorsally; hind tibia densely erect setose; [middle lobe of mesoscutum evenly setose]	***X.comatus* (Wesmael, 1835)**
–	Antenna of ♀ with 28–34 segments, 1.3–1.5× longer than body; apex of metasoma of ♀ yellow; hind femur smooth and with medium-sized setae dorsally; hind tibia adpressed setose	**7**
7	Lateral mesoscutal lobes glabrous medially, only laterally with long setae; notauli completely developed on mesoscutal disc; vein 3-CU1 of fore wing about as long as vein CU1b; antenna of ♀ with 28–31 segments; sixth metasomal tergite largely dark brown; second tergite finely striate; vein m-cu of fore wing postfurcal	***X.aciculatus* (Thomson, 1895)**
–	Lateral mesoscutal lobes largely setose medially; notauli largely absent on mesoscutal disc; vein 3-CU1 of fore wing distinctly longer than vein CU1b; antenna of ♀ with 32–34 segments; sixth metasomal tergite yellow; second tergite coarsely striate; vein m-cu of fore wing antefurcal	***X.setosiscutum* van Achterberg, sp. nov.**

### ﻿Key to Japanese species of the genus *Xynobius* Foerster

**Notes.** The number of included species for Japan is based on the list by Yu et al. (2016); only *Xynobiussapporanus* (Fischer, 1963) is added as a new combination. Types of *X.macrocerus*, *X.sapporanus* and *X.subparallelus* have been examined.

**Table:** 

1	Scutellum coarsely punctate; [pterostigma subparallel-sided; antenna with 50–54 segments]	***X.japanus* (Fischer, 1963)**
–	Scutellum smooth	**2**
2	Middle mesoscutal lobe evenly setose	**3**
–	Mesoscutal lobes glabrous medially, only along notauli with some long setae	**4**
3	Fore wing membrane with a large Y-shaped dark brown patch below para- and pterostigma (smaller in ♂); notauli largely impressed on mesoscutal disc; body black	***X.sapporanus* (Fischer, 1963) comb. nov.**
–	Fore wing membrane hyaline, without dark patch; notauli largely absent on mesoscutal disc; body dark brown or yellowish brown	***X.subparallelus* Han & van Achterberg, sp. nov.**
4	Propodeum largely sculptured and without medio-longitudinal carina	**5**
–	Propodeum largely smooth or anteriorly with a medio-longitudinal carina	**7**
5	Vein m-cu of fore wing interstitial or antefurcal; second metasomal tergite smooth; [antenna with about 37 segments]	***X.laticella* (Tobias, 1998)**
–	Vein m-cu of fore wing postfurcal and/or second tergite sculptured	**6**
6	Second metasomal tergite superficially rugulose; antenna with about 25 segments and slightly longer than body	***X.kamikochiensis* (Fischer, 1963)**
–	Second tergite smooth; antenna with 32 or 33 segments and about 1.3× longer than body	***X.claricoxa* (Fischer, 1963)**
7	Pterostigma wide elliptical and distinctly narrowed distally; antenna with 29–31 segments; second metasomal tergite largely longitudinally rugose	***X.kotenkoi* (Fischer, 1998)**
–	Pterostigma subparallel-sided and slightly widened distally; antenna with 50–53 segments; second tergite smooth	***X.macrocerus* (Thomson, 1895)**

## Supplementary Material

XML Treatment for Xynobius

XML Treatment for Xynobius subparallelus

XML Treatment for Xynobius setosiscutum
